# Wet-chemical synthesis and applications of non-layer structured two-dimensional nanomaterials

**DOI:** 10.1038/ncomms8873

**Published:** 2015-08-25

**Authors:** Chaoliang Tan, Hua Zhang

**Affiliations:** 1 School of Materials Science and Engineering, Nanyang Technological University, 50 Nanyang Avenue, Singapore 639798, Singapore

## Abstract

Non-layer structured nanomaterials with single- or few-layer thickness have two-dimensional sheet-like structures and possess intriguing properties. Recent years have seen major advances in development of a host of non-layer structured ultrathin two-dimensional nanomaterials such as noble metals, metal oxides and metal chalcogenides. The wet-chemical synthesis has emerged as the most promising route towards high-yield and mass production of such nanomaterials. These nanomaterials are now finding increasing applications in a wide range of areas including catalysis, energy production and storage, sensor and nanotherapy, to name but a few.

## Abstract (web summary)

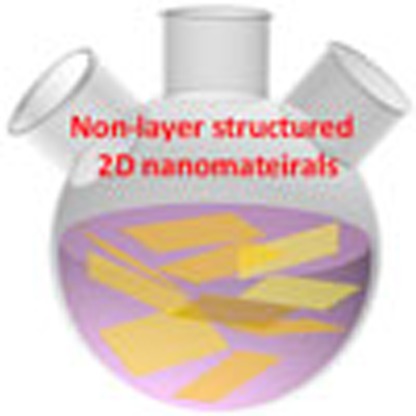
There is currently intensive research underway into the development of non-layer structured two dimensional nanomaterials. Here, Zhang *et al.* review the research progress on the most promising wet-chemical synthesis methods as well as a wide range of applications of this unique class of materials.

Since the exfoliation and identification of graphene in 2004 (ref. 1), layered ultrathin two-dimensional (2D) nanomaterials have been the subject of intensive study over the last decade^2^,^3^,^4^,^5^. Ultrathin 2D nanomaterials are sheet-like structures with single- or few-layer thickness (typically less than 5 nm), but lateral size larger than 100 nm or even up to tens of micrometres. Till now, besides graphene^1^,^2^,^6^, a large number of graphene-like ultrathin 2D nanomaterials, such as transition metal dichalcogenides, layered metal oxides, transition metal carbides and layered-double hydroxides, have been prepared via various methods^3^,^4^,^5^,^7^,^8^,^9^,^10^,^11^,^12^. Owing to the ultrahigh specific surface area and strong quantum confinement of electrons in two dimensions, these ultrathin 2D nanomaterials display many unconventional physical, optical, chemical and electronic properties. They have also shown great potential in various applications such as electronic devices^1^,^13^,^14^,^15^, catalysis^16^,^17^,^18^, energy storage and conversion^19^,^20^, sensing^21^,^22^ and biomedicine^23^.

Layered compounds are those that possess strong lateral chemical bonding in planes but display weak van der Waals interaction between planes. One typical example is graphite that consists of weakly stacked graphene sheets forming three-dimensional (3D) bulk crystals. However, many other materials form atomic bonding in three dimensions (for example, metals), reflecting the non-layered nature of their bulk crystals. Inspired by the layered ultrathin 2D crystals, one can also anticipate that controlled synthesis of non-layer structured 2D materials may bring up some unique properties and advanced functions that cannot be achieved for their counterparts in other dimensionalities. A host of non-layer structured ultrathin 2D nanomaterials, such as noble metals (for example, Au, Pd and Rh), metal oxides (for example, TiO_2_, WO_3_, CeO_2_, In_2_O_3_, SnO_2_, Fe_2_O_3_ and so on) and metal chalcogenides (for example, PbS, CuS, CuSe, SnSe, ZnSe, ZnS, CdSe and so on), have been prepared over the last few years; almost all of these non-layer structured ultrathin 2D nanomaterials are synthesized using wet-chemical synthesis approaches. Expectedly, the synthesized 2D nanomaterials offer some unique advantages in comparison with their counterparts in other dimensionalities and hold great promises in a variety of applications, such as catalysis, supercapacitors, photodetectors and photothermal therapy. Although many reviews on ultrathin 2D nanomaterials are available in the literature^2^,^3^,^4^,^5^,^6^,^7^,^9^,^10^,^11^,^12^, almost all of them focus on the layered 2D crystals. Therefore, a timely, comprehensive review on non-layer-structured 2D nanomaterials is of great importance for the future study.

In this review, we aim to give an overview on the recent progress of wet-chemical synthesis and applications of non-layer structured 2D nanomaterials such as metals, metal oxides and metal chalcogenides. We first introduce various types of wet-chemical synthesis strategies for preparation of non-layer structured 2D nanomaterials including 2D-templated synthesis, hydro/solvothermal synthesis, self-assembly of low-dimensional nanocrystals and soft colloidal templated synthesis. Then some promising applications, especially in catalysis, of the synthesized 2D nanomaterials are briefly described, with emphasis on those with excellent performance. Finally, a summary of current research status and progress, along with some personal perspectives on the challenges and future research directions in this promising area, are given.

## Wet-chemical synthesis method and structure characterization

Till now, a variety of synthetic strategies, such as mechanical exfoliation^1^,^24^,^25^,^26^, liquid exfoliation^5^,^27^,^28^,^29^,^30^, ion-intercalation and exfoliation^5^,^31^,^32^,^33^,^34^,^35^, chemical vapour deposition (CVD)^36^,^37^,^38^,^39^,^40^ and wet-chemical synthesis^41^,^42^,^43^,^44^,^45^,^46^,^47^,^48^, have been developed for preparation of ultrathin 2D nanomaterials. The comparison of some typical methods for synthesis of ultrathin 2D nanomaterials is summarized in Table 1.

The mechanical exfoliation technique that was first used to produce graphene is the typical approach for producing single- or few-layer nanosheets from their corresponding layered bulk crystals^1^,^24^,^25^,^26^. The pristine 2D crystals (for example, graphene and MoS_2_) obtained by this approach often show high quality and large lateral size, which are ideal for investigation of their intrinsic properties and fabrication of electronic devices. However, the low throughput at the current form limits their practical applications. Consequently, as a promising alternative, liquid exfoliation by direct sonication of layered bulk crystals in solvents or surfactant/polymer solutions was developed to produce ultrathin 2D nanomaterials in high yield and large scale^5^,^27^,^28^,^29^,^30^. However, the yield of single-layer sheets produced by this method is low and it is hard to separate the single-layer sheets from the solution. Alternatively, the ion-intercalation and exfoliation method was developed towards the high-yield and large-scale production of single-layer nanosheets, such as graphene, MoS_2_, TiS_2_, TaS_2_, WS_2_, ZrS_2_, *h*-BN and so on^5^,^31^,^32^,^33^,^34^,^35^. However, the 2D nanosheets obtained by this method might possess many defects and relatively small lateral size, and this approach is also very sensitive to water and oxygen. It should be noted that all aforementioned exfoliation methods are only applicable to those materials whose bulk crystals are layered.

In addition to these exfoliation methods, CVD method is another appealing strategy for growth of high-quality single-crystalline 2D sheets on substrates with scalable size, controllable thickness and excellent electronic properties^36^,^37^,^38^,^39^,^40^. However, the CVD method suffers from the requirement of high temperature, high vacuum and specific substrates. Wet-chemical synthesis has been emerging as a very promising alternative towards the high-yield, low-cost and mass production of all types of ultrathin 2D nanosheets in the solution phase^41^,^42^,^43^,^44^,^45^,^46^,^47^,^48^. Particularly, almost all the non-layer structured ultrathin 2D nanomaterials, such as metals, metal chalcogenides and metal oxides, can be synthesized using wet-chemical synthesis methods because of their non-layered nature.

Meanwhile, along with the development of nanotechnology, some useful and powerful techniques have been identified or developed for characterization of these non-layer structured 2D nanomaterials. For example, the commonly used techniques for characterization of traditional nanomaterials, such as scanning electron microscopy (SEM), transmission electron microscopy, X-ray diffraction, atomic force microscopy, Raman spectroscopy, energy-dispersive X-ray spectroscopy and X-ray photoelectron spectroscopy, have been used to characterize the size, thickness, crystallinities, exposed crystal facets, compositions and structures of these ultrathin 2D nanomaterials. The X-ray absorption fine structure spectroscopy is another powerful technique to study the local atomic geometry and chemical state of atoms of one specific element in the ultrathin 2D materials, allowing for understanding the defects, vacancies and doping effects^12^. In this section, we focus on several main wet-chemical synthesis methods including 2D-templated synthesis, hydro/solvothermal synthesis, self-assembly of low-dimensional nanocrystals and soft colloidal templated synthesis. We give examples of synthesis of non-layer-structured 2D nanomaterials along with their characterization by various techniques. The summary of the dimension, synthesis methods and used characterization techniques of these non-layer-structured 2D nanomaterials are shown in Table 2.

### 2D-templated synthesis

**Figure 1:**
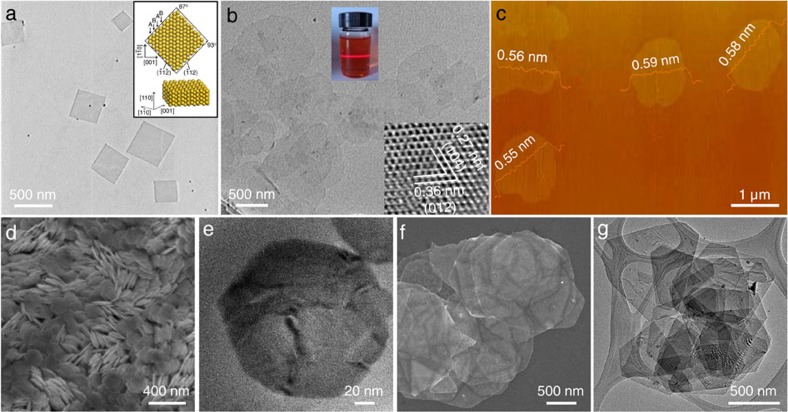
2D nanosheets synthesized using the 2D-templated synthesis method. (**a**) TEM images of *hcp* AuSSs. Inset: crystallographic models for a typical AuSS with its basal plane along the [110]*h* zone axis, showing ABAB stacking along the [001]*h* direction. Adapted from ref. 51 (**b**) TEM image of *α*-Fe_2_O_3_ nanosheets. Inset: HRTEM image and Tyndall effect of *α*-Fe_2_O_3_ nanosheets. Adapted, with permission, from ref. 55 (copyright 2014 American Chemical Society). (**c**) Atomic force microscopy (AFM) image of *α*-Fe_2_O_3_ nanosheets. Adapted, with permission, from ref. 55. (Copyright 2014, American Chemical Society). (**d**) SEM and (**e**) TEM images of CuInS_2_ nanosheets. Reproduced, with permission, from ref. 56 (© 2014 John Wiley & Sons Inc). (**f**) TEM and (**g**) SEM images of CuSe and Cu_2−*x*_Se nanosheets, respectively. Reproduced, with permission, from ref. 57 (© 2014 John Wiley & Sons Inc).

Templated synthesis methods have been widely used for growth of anisotropic nanocrystals (for example, nanowires)^49^,^50^, in which the crystal growth can be confined in specific dimension. Non-layer structured 2D nanomaterials can be synthesized using prepared 2D nanomaterials as templates^51^,^52^,^53^,^54^,^55^,^56^,^57^,^58^. As a typical example, our group prepared Au square nanosheets (AuSSs) with thickness of ∼2.4 nm (∼16 Au atomic layers) and size of 200–500 nm by reducing HAuCl_4_ with 1-amino-9-octadecene on graphene oxide template (Fig. 1a)^51^. As can be seen, this AuSS crystal owns unique hexagonal-close packed (*hcp*) phase rather than the common face-centred cubic (*fcc*) phase. This is the first time that the pure *hcp* Au nanostructure, which can be stable under ambient conditions, has been synthesized. Furthermore, thick Au sheets can be obtained via a secondary growth by using this *hcp* AuSS as the seed and a phase transformation from *hcp* to *fcc* was observed during the secondary growth process^52^. The centre of resultant thick Au sheets has an alternating *hcp/fcc* structural domain, while the edge site is defect-free *fcc* structure. Significantly, our group has also demonstrated recently that the further coating of Ag thin layer on the *hcp* AuSSs could induce the phase transformation to obtain (100)_f_-oriented *fcc* Au@Ag core–shell square sheets or (110)_h_/(101)_f_-oriented *hcp*/*fcc* Au@Ag square sheets^53^. Similarly, the further growth of Pt or Pd thin layer on AuSSs also could induce the phase transformation from the *hcp* to *fcc* structures to form core–shell Au@Pt or Au@Pd nanoplates^54^. However, unlike the coating of Ag, the coating of Pt or Pd on *hcp* AuSSs mainly leads to the formation of *fcc* Au@Pt or Au@Pd rhombic nanoplates with (101)_f_ orientation, although a little amount of *fcc* (100)_f_-oriented Au@Pt and Au@Pd square nanoplates can also be observed. It is believed that the large lattice mismatch between Pt or Pd and Au compared with Ag is responsible for the formation of unprecedented (101)_f_-oriented core–shell nanoplates. More interestingly, the complete phase transformation of AuSSs from *hcp* to *fcc* structures was realized via the simple ligand exchange under ambient conditions at room temperature^53^. By simply replacing the oleylamine capped on AuSSs with thiol molecules, the *hcp* AuSSs can be transformed into (100)_f_-oriented *fcc* AuSSs (ref. 53). Interestingly, Wei and coworkers^55^ recently developed a CuO nanoplate-templated method to synthesize freestanding half-unit-cell *α*-Fe_2_O_3_ nanosheets. The layered iron hydroxide nanosheets were first prepared on the CuO template surface. Then the CuO template was slowly etched away to get the freestanding iron hydroxide nanosheets. Finally, an annealing treatment was carried out to transform the iron hydroxide sheets into freestanding *α*-Fe_2_O_3_ nanosheets with size of up to ∼1 μm and thickness of 0.55–0.59 nm (Fig. 1b,c).

The used templates (for example, graphene oxide and CuO) in the aforementioned examples are inert during the synthesis process. Recently, our group reported the use of semiconductor nanosheets (for example, CuSe and CuS) as reactive templates to prepare novel 2D nanostructures^56^,^57^. For example, by using the synthesized CuS nanosheet with thickness of ∼4.8 nm as the reactive template, a series of copper-based ternary and quaternary chalcogenide nanosheets including CuInS_2_, CuIn_*x*_Ga_1−*x*_S_2_ and Cu_2_ZnSnS_4_ were successfully prepared via the cation exchange process (Fig. 1d,e)^56^. The shape and size of nanosheets did not undergo significant changes during the transformation process. All the resultant ternary and quaternary semiconductor nanosheets have uniform size, shape and thickness. In addition, our group also demonstrated that semiconductor nanosheets (for example, Cu_2−*x*_Se and Cu_1.97_S) with different crystal phases can be synthesized via phase transformation by using as-prepared nanosheets (for example, CuSe and CuS) as templates^57^. Briefly, the synthesized hexagonal-phased CuSe nanosheets were transformed into cubic-phased Cu_2−*x*_Se nanosheets by simply heating treatment in presence of Cu^I^ cations. Both the CuSe and Cu_2−*x*_Se nanosheets have similar morphologies with size up to micrometre and thickness of ∼5 nm (Fig. 1f,g). Notably, a similar transformation from the ultrathin hexagonal-phased CuS nanosheets to cubic-phased Cu_1.97_S nanosheets can also be realized by using this method. In addition, Li and co-workers demonstrated the synthesis of NiO nanosheets with size of up to micrometre and thickness of <2 nm from layered *α*-Ni(OH)_2_ nanosheets through a simple annealing treatment^58^.

### Hydro/solvothermal synthesis

**Figure 2:**
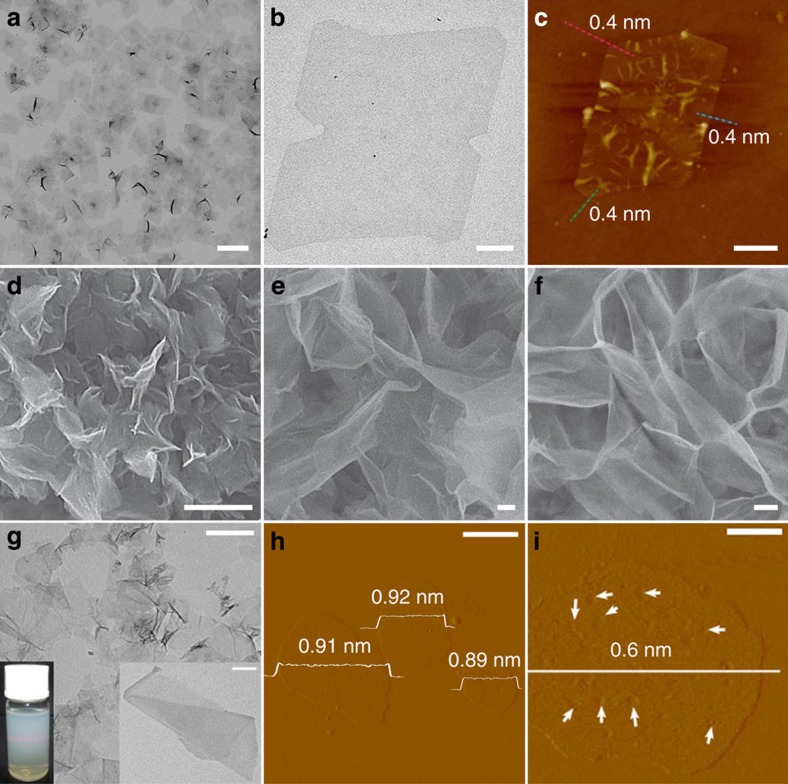
2D nanosheets synthesized using the hydro/solvothermal synthesis method. (**a**,**b**) TEM image of the PVP-capped Rh nanosheets, and (**c**) AFM image of a bare Rh nanosheet. Adapted from ref. 59. (**d**) SEM images of 2D nanosheets of TiO_2_, ZnO (**e**) and Co_3_O_4_ (**f**; scale bars, 200 nm). Adapted from ref. 60. (**g**) TEM image of ZnSe single layers (scale bar, 500 nm). Inset: the enlarged TEM image (scale bar, 100 nm) and Tyndall effect of ZnSe single layers. Adapted from ref. 61. (**h**) AFM image ZnSe single layers (scale bar, 500 nm). Adapted from ref. 61. (**i**) AFM image of ultrathin surface-pitted CeO_2_ sheets (scale bars, 100 nm). Adapted from ref. 62.

Hydro/solvothermal synthesis is another typical method that has been widely used for synthesis of non-layer structured 2D nanomaterials^59^,^60^,^61^,^62^,^63^,^64^,^65^,^66^,^67^. As a typical example, Li and co-workers demonstrated the synthesis of poly(vinylpyrrolidone) (PVP)-supported single-layer rhodium (Rh) nanosheets with edge length of 500–600 nm and thickness of ∼0.4 nm via a facile solvothermal route (Fig. 2a–c)^59^. This is the first time for synthesis of single-layer atomic metal nanostructures in the liquid phase. In addition, Dou and co-workers developed a generalized solvothermal method for the synthesis of a series of metal oxide nanosheets, including TiO_2_, ZnO, Co_3_O_4_, WO_3_, Fe_3_O_4_ and MnO_2_ (Fig. 2d–f)^60^. The size of the TiO_2_ nanosheets is ∼200 nm, while the sizes of ZnO, Co_3_O_4_ and WO_3_ are up to 1–10 μm. Similarly, the thickness of these metal oxide nanosheets varied between 1.6 and 5.2 nm, corresponding to 2–7 stacking layers of the monolayer.

In addition, Xie and co-workers used hydro/solvothermal method to synthesize several kinds of non-layer structured 2D nanosheets including ZnSe, ZnS, CeO_2_, In_2_O_3_, SnO_2_, Co_9_Se_8_ and Co_9_S_8_-oleylamine hybrid^61^,^62^,^63^,^64^,^65^,^66^. For instance, to synthesize ZnSe and ZnS nanosheets, the lamellar organic–inorganic intermediates, for example, (Zn_2_Se_2_)(*n*-propylamine) and (Zn_2_S_2_)(*n*-propylamine), were first synthesized using a solvothermal method^61^. Then the hybrid intermediates were exfoliated by sonication to get freestanding ultrathin ZnSe and ZnS nanosheets, respectively. The resultant ZnSe nanosheets possess a lateral size of ∼500 nm and thickness of ∼0.9 nm (Fig. 2g,h). Similarly, Zhang and co-workers reported the preparation of CdS nanosheets with size of 300–800 nm and thickness of ∼4 nm via a similar strategy by using the diethylenetriamine (DETA) as the surfactant^67^. Moreover, Xie and co-workers reported that atomically thin CeO_2_ sheets with surface-confined pits could be prepared by a hydrothermal method combined with the subsequent thermal annealing treatment^62^. Briefly, CeCO_3_OH sheets were first synthesized from cerium chloride using sodium oleate as the surfactant via a hydrothermal method. Then the intermediate sheets were transformed into ultrathin CeO_2_ sheets with surface-confined pits having a thickness of ∼0.6 nm via a thermal annealing treatment (Fig. 2i). Besides, ultrathin In_2_O_3_ porous sheets with rich oxygen vacancies were also successfully synthesized via a similar hydrothermal method with subsequent thermal annealing process^63^.

### Self-assembly of low-dimensional nanocrystals

Self-assembly of low-dimensional nanocrystals (for example, nanoparticles (NPs) and nanowires) is also a promising alterative way to prepare ultrathin 2D nanomaterials^68^,^69^,^70^,^71^,^72^,^73^. Generally, the fusion of low-dimensional nanocrystals occurs during the assembly process to form 2D nanomaterials. As a typical example, Weller and co-workers used a typical self-assembly strategy, that is, 2D-oriented attachment, to prepare ultrathin PbS nanosheets with size of 0.8–2 μm and thickness of ∼2.2 nm from tiny PbS NPs (ref. 68). The ordered and densely packed ligand layers of oleic acid on {100} surface of PbS NPs can drive the 2D-oriented attachment of PbS NPs to form large sheets. The chlorine-containing solvents in the initial nucleation and growth process of nanocrystals are also essential in the orientated attachment process. The 2D-oriented attachment is an entropy-driven crystal growth and reconstruction process, which minimizes high-surface-energy facets and interfaces between NPs, leading to the coalescence of NPs to form a large crystal. The formation mechanism was further confirmed by Wang and co-workers using the *in situ* small and wide-angle synchrotron X-ray scattering on the same spot of sample under pressure coupled with transmission electron microscopy^69^. Recently, single-crystalline WO_3_ nanosheets with thickness of 4–5 nm and lateral size up to micrometre have also been constructed via 2D-oriented attachment of tiny WO_3_ nanocrystals with size of 4–5 nm (ref. 70). As an alternative, Lu and co-workers demonstrated the self-assembly of Au nanoclusters into ultrathin nanosheets with single-cluster thickness^71^. The as-prepared Au_15_ clusters in a mixed solvent of dibenzyl ether and liquid paraffin were simply annealed at 140 °C to evaporate solvents to form assembled Au sheets at the interface. The assembled Au sheet with width of ∼300 nm and length of 200–1,000 nm is consistent of Au clusters rather than a single-crystal structure.

In addition to NPs, the assembly of nanowires into ultrathin 2D nanosheets has been also achieved recently^72^,^73^. For example, Yao and co-workers reported the synthesis of Eu_2_O_3_ nanosheets from the assembly of Eu_2_O_3_ nanowires with diameter of ∼1.5 nm (ref. 72). The Eu_2_O_3_ nanowire bundles in 1,5-pentanediol were transferred into water solution and soaked for different time. Then they were assembled in sheet-like porous structures after 3–5 min soaking, and further transformed to rectangle nanosheets when the soaking time increased to 1 h. The thickness of resultant sheets is ∼3.8 nm. The lateral size of Eu_2_O_3_ sheets can be tuned from several hundreds of nanometres to 10 μm by simply controlling the soaking time of nanowires. With similar strategy, Acharya *et al.*^73^ demonstrated the assembly of ultrathin PbS nanowires into 2D nanosheets with regular rectangular shape of 200–250 in width, 3–20 μm in length and ∼1.8 nm in thickness. The as-prepared PbS nanowires with diameter of ∼1.8 nm were assembled and fused together to form sheet structure via a collective coalescence approach at the air–water interface.

### Soft colloidal templated synthesis and other methods

**Figure 3:**
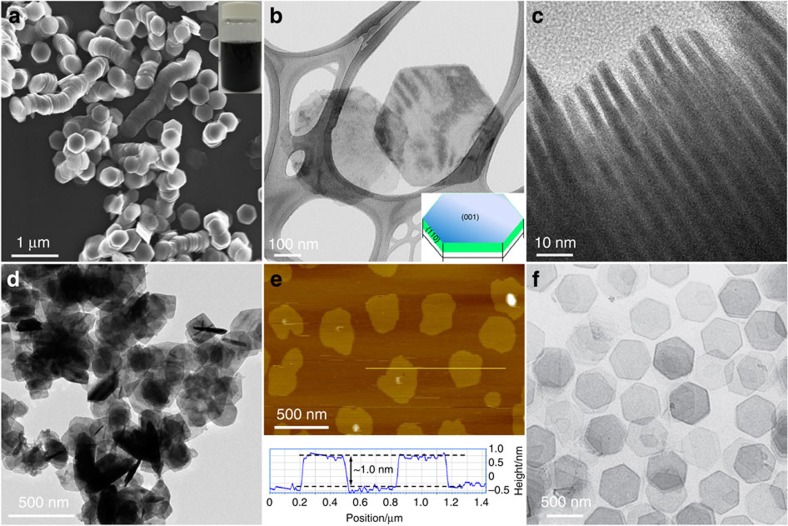
2D nanosheets synthesized using soft colloidal templated synthesis and other methods. (**a**) SEM image of ultrathin CuS nanosheets. Inset: photograph of the colloid solution of CuS nanosheets. Adapted from ref. 77. TEM images of ultrathin CuS nanosheets with (**b**) lying flat and (**c**) standing on the TEM grids. Inset in **b**: scheme of an ultrathin CuS nanosheet. Adapted from ref. 77. (**d**) TEM and (**e**) AFM images of SnSe nanosheets. Adapted, with permission, from ref. 78 (Copyright 2013 American Chemical Society). (**f**) TEM image of Pd nanosheets. Adapted from ref. 81.

Generally, soft colloidal templated synthesis represents a type of oil phase-based methods^74^,^75^, in which the long-chain oleyl amine and/or oleic acid surfactants are used as the soft colloidal templates for directing the crystal growth. Recently, the soft colloidal templated synthesis has been employed for preparation of ultrathin 2D nanocrystals, especially for semiconductors^76^,^77^. In 2009, Hyeon and co-workers used this method to synthesize wurtzite ultrathin CdSe nanosheets^76^. It is supposed that intermediate lamellar complexes composed of 2D arrays of CdCl_2_ and alkyl amine or/and oleic acid were first obtained, in which the alkyl amine or/and oleic acid served as the soft colloidal template. Then the intermediate lamellar complex further reacted with the Se source to form stacked CdSe nanosheets. It was found that well-separated ultrathin CdSe nanosheets can be obtained by using the mixture of alkyl amine and oleic acid as the soft template in contrast to the use of single long-chain surfactant (for example, alkyl amine or oleic acid). The resultant single-layer CdSe sheets possess length of 200–300 nm, width of ∼100 nm and thickness of ∼1.4 nm. Recently, our group reported the generalized synthesis of ultrathin metal sulphide nanocrystals by using the soft colloidal templated strategy^77^. Ultrathin CuS nanosheets with thickness of ∼3.2 nm (two unit cells) were prepared in gram amount (Fig. 3a–c). The resultant CuS nanosheets have regular hexagonal shape with lateral size up to ∼453 nm (Fig. 3a,b).

Some other wet-chemical methods have also been developed for synthesis of ultrathin 2D nanostructures, which cannot be categorized into the aforementioned methods^78^,^79^,^80^,^81^. As a typical example, Wang and co-workers presented a one-pot synthetic method for synthesis of single-layer SnSe nanosheets in oil phase^78^. The 1,10-phenanthroline was used as the morphology control agent, which played a crucial role in controlling the morphology of SnSe nanocrystals. The obtained SnSe nanosheets have lateral size of ∼300 and thickness of ∼1.0 nm (Fig. 3d,e). As another example, Yan and co-workers synthesized several ultrathin rare-earth oxide nanosheets in the oil phase in presence of ionic liquid salts^79^. Two types of Gd_2_O_3_ nanosheets with widths of 100 and 200 nm and thicknesses of 0.35 and 0.65 nm, respectively, were synthesized. Besides, this general method can also be extended to prepared Ho_2_O_3_ and Y_2_O_3_ nanosheets with thicknesses less than 1 nm. Very recently, Xie and co-workers demonstrated that the synthesis of single-layer *β*-Co(OH)_2_ nanosheets can be achieved by a simple and green approach under ambient atmospheric conditions using cobalt chloride and aminoethanol as precursors^80^. As an interesting example, Zheng and co-workers reported a CO-confined growth method to synthesize freestanding ultrathin hexagonal Pd nanosheets with thickness less than 10 atomic layers (∼1.8 nm) and controllable edge length from 20 to 160 nm (Fig. 3f)^81^. The use of CO as the capping agent is essential for the growth of Pd nanocrystals, which were confined in 2D because of its strong adsorption on the basal (111) planes of Pd nanosheets.

## Applications of the non-layer structured 2D nanomaterials

Owing to their large lateral size and ultrathin thickness, 2D nanomaterials possess ultrahigh specific surface area^82^, and thus are ideal candidates for surface-active applications. For instance, ultrathin 2D nanomaterials have been proved to be fascinating platforms for engineering high-efficient catalysts for various kinds of catalytic applications^83^,^84^. It was found that some of the synthesized ultrathin 2D nanosheets have excellent activities in a number of catalytic processes. In addition, the ultrahigh surface area of 2D nanomaterials also makes them very promising electrode materials for supercapacitors and photodetectors. In this section, we will discuss the synthesized ultrathin 2D nanomaterials for various applications including catalysis, supercapacitors, photodetectors and photothermal therapy, with emphasis on those materials with excellent performance. The summary of the non-layer structured 2D nanomaterials for different applications is made in Table 2.

### Organic catalytic reactions

**Figure 4:**
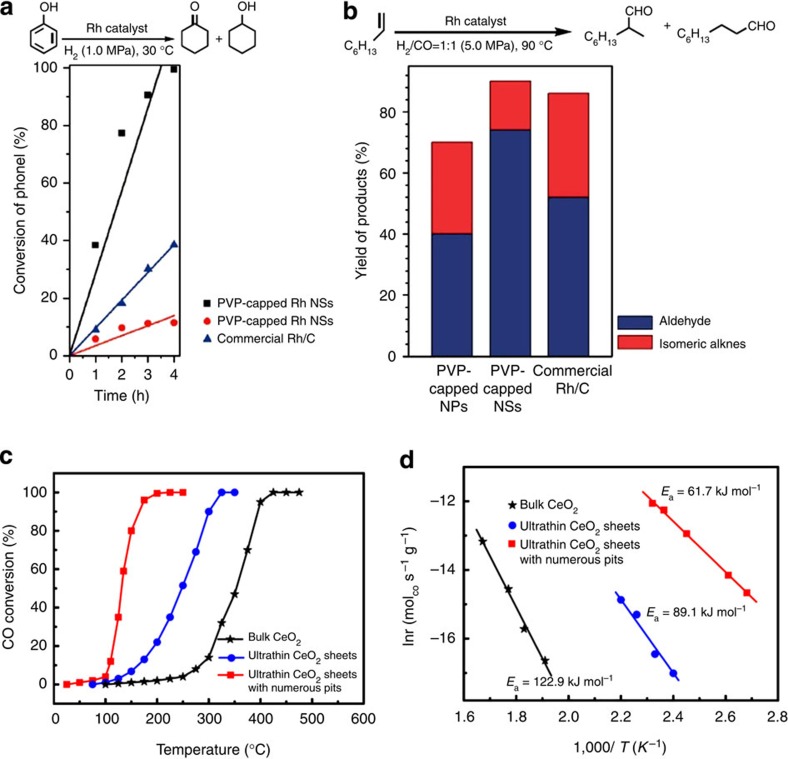
Catalytic activities of Rh and CeO_2_ nanosheets. (**a**) Hydrogenation of phenol and (**b**) hydroformylation of 1-octene. Adapted from ref. 59. (**c**) The reaction temperature-dependent catalytic activities of CeO_2_-based catalysts for CO oxidation (experimental error: ±3%), and (**d**) the corresponding Arrhenius plot for the three CeO_2_-based samples (experimental error: ±3%). Adapted from ref. 62.

Li and co-workers demonstrated that the single-layer Rh nanosheets could be a high-efficient catalyst for catalytic hydrogenation and hydroformylation reactions (Fig. 4a,b)^59^. Hydrogenation of phenol and hydroformylation of 1-octene were used as probe reactions to investigate the catalytic properties of PVP-capped Rh nanosheets. The PVP-capped Rh sheets gave >99.9% conversion within 4 h at near room temperature (30 °C) under low H_2_ pressure for hydrogenation of phenol. The catalytic activity of conversion at 1 h is seven and four times higher than those of PVP-capped Rh NPs and commercial Rh/C, respectively. In the hydroformylation of 1-octene, the PVP-capped Rh nanosheets also exhibited both superior catalytic activity and selectivity towards the target product (aldehyde) under mild reaction conditions. The excellent catalytic properties make the ultrathin Rh nanosheets a promising catalyst for catalytic organic reactions. It is well known that the surface atom ratio is a key factor in catalysis. Owing to its single-layer feature, the ratio of the surface Rh atom in Rh nanosheets is 100%. As a result, the largely enhanced catalytic activity could be ascribed to the full exposure of Rh atoms in the Rh nanosheets.

### Electrocatalytic oxidation of formic acid

It was found that the Pd nanosheets could be highly efficient in electrocatalysis for oxidation of formic acid^81^. Owing to its high specific surface area, the measured electrochemically active surface area of Pd nanosheets with edge length of 41 nm was calculated to be as high as 67 m^2 ^g^−1^, which is slightly lower than the theoretically calculated maximum surface area (∼100 m^2 ^g^−1^). The experimental results indicated that the Pd nanosheets have excellent electrocatalytic activity for the oxidation of formic acid. The current density of Pd nanosheets for formic acid oxidation was measured to be 1,380 mA mg^−1^ at 0.14 V, which is much higher (∼2.5 times) than that of the commercial palladium black catalyst.

### Photocatalytic water splitting

The ultrathin ZnSe nanosheet has been identified to be a promising catalyst for photoelectronchemical (PEC) solar water splitting^61^. By simply spin-coating ZnSe single layers, few atom-thick ZnSe sheets and bulk ZnSe on a polyethylene terephthalate substrate, flexible and transparent photoelectrodes were fabricated for water photocatalysis. All ZnSe-based photoelectrodes gave a very low current density (<5 μA cm^−2^) under the scan from −0.4 to 0.8 V in dark. Note that the single-layer ZnSe sheet-based photoelectrode showed photocurrent density of 2.14 mA cm^−2^ at 0.72 V under the irradiation of 300 W Xe lamp, which is higher than that of all other ZnSe-based photoelectrodes, and roughly 195 times higher than that of the bulk counterpart. Moreover, the ZnSe sheets also exhibited strong intensity and frequency-dependent photocurrent density. Compared with the ZnSe quantum dots with average size of 3–4 nm, the single-layer ZnSe nanosheets also showed much higher photocurrent density at 0.72 V, further confirming its strikingly efficient PEC water splitting. Similarly, it was found that the ultrathin porous In_2_O_3_ nanosheets with rich oxygen vacancy also presented superior performance compared with ultrathin porous In_2_O_3_ sheets with poor oxygen vacancy and bulk In_2_O_3_ when they were used as photoelectrodes in visible light PEC cells^63^.

Recently, Zhang and co-workers demonstrated that the ultrathin CdS nanosheets could be used as an efficient photocatalyst for hydrogen generation^67^. For comparison, the photocatalytic activities of some other CdS-based samples including CdS-DETA hybrid nanosheets, CdS nanosheet-based aggregates and CdS NPs were also studied. The average hydrogen production rate of CdS nanosheets reached 41.1 mmol h^−1 ^g^−1^, which is strikingly higher than that of the CdS-DETA hybrid nanosheets (7.5 mmol h^−1 ^g^−1^), CdS nanosheet-based aggregates (6.7 mmol h^−1 ^g^−1^), and CdS NPs (negligible). The apparent quantum efficiency of ultrathin CdS nanosheets was determined to be 1.38% at 420 nm. It was found that loading of small amount of PbS NPs (1 wt%) on CdS nanosheets can further significantly enhance the quantum efficiency to as high as 9.62%. Note that no obvious decrease in the H_2_ production rate was observed even after 12-h H_2_ evolution, indicating the good stability of catalyst.

### Catalytic CO oxidation

Xie and co-workers reported that the ultrathin CeO_2_ nanosheets with ∼20% surface pits could be used as a high-efficiency catalyst for CO oxidation^62^. The pit-confined CeO_2_ nanosheets presented 50% conversion of CO to CO_2_ at 131 °C, which is much lower than that of the intact CeO_2_ sheets (248 °C) and bulk counterpart (345 °C; Fig. 4c). At 131 °C, the conversion ratio of intact CeO_2_ sheets and bulk crystals is only 3.2 and 0.89%, respectively. The temperature for these CeO_2_ nanosheets with surface pits used to completely convert CO to CO_2_ in the CO oxidation is ∼200 °C, which is also lower than those for CeO_2_ nanosheets (325 °C) and bulk materials (425 °C; Fig. 4c). It is worth pointing out that the catalytic property of these ultrathin pit-confined CeO_2_ nanosheets is the best so far among all reported CeO_2_-based catalysts for CO oxidation. In addition to the conversion temperature, the apparent activation energy of catalyst is another important parameter used for evaluation of CO oxidation. Significantly, the apparent activation energy of CeO_2_ nanosheets with numerous surface pits is 61.7 kJ mol^−1^, while that for CeO_2_ nanosheets and bulk counterpart is 89.1 and 122.9 kJ mol^−1^, respectively (Fig. 4d). The structural analysis of CeO_2_ nanosheets with surface pits revealed that the Ce sites have average coordination number of 4.6, indicating the unsaturated coordination nature of Ce at pit sites. The superior catalytic performance of the CeO_2_ nanosheets with numerous surface pits is attributed to the unsaturated-coordinated pit-surrounding Ce sites. According to the theoretical calculation, the four-coordinated and five-coordinated Ce sites are the more active sites for CO catalytic oxidation. In this case, The CeO_2_ sheets with these sites possess strikingly lower apparent activation energy in comparison with the other two CeO_2_ samples, resulting in the much lower conversion temperature for CO oxidation.

In addition, the same group demonstrated that the ultrathin SnO_2_ nanosheets with thickness of 0.6 nm also present excellent performance for the CO catalytic oxidation^64^. The ignition temperature used for single-layer SnO_2_ nanosheets with conversion ratio of ∼10% for CO oxidation is 124 °C, while for other counterparts it is much higher, that is, 203 °C for 1.9-nm-thick SnO_2_ nanosheets, 270 °C for SnO_2_ NPs and 360 °C for bulk SnO_2_. More importantly, the conversion temperature of 0.6-nm-thick SnO_2_ nanosheets is 165 °C, which is much lower than that of the 1.9-nm-thick SnO_2_ sheets (330 °C), SnO_2_ NPs (390 °C) and bulk SnO_2_ (475 °C). Similarly, the apparent activation energy of these samples also gives the similar tendency. The outstanding performance of 0.6-nm-thick SnO_2_ nanosheets could be ascribed to the extremely high specific surface area. Both the Sn and O atoms at surface sites have unsaturated coordination numbers that are favourable for CO catalytic oxidation, which is further confirmed by the density functional theory (DFT) calculations^64^.

### Catalytic conversion of CO_2_ to CH_4_

The conversion of CO_2_ to CH_4_ fuel is of great importance to address both of the global warming and the energy-shortage problems^85^. As a typical example, Zou and co-workers presented that the ultrathin WO_3_ nanosheet could be a good photocatalyst for the reduction of CO_2_ to CH_4_ with H_2_O (ref. 70). It was found that CO_2_ and H_2_O can react with the photogenerated electrons and holes in the WO_3_ nanosheets under visible light irradiation to produce CH_4_ and O_2_ in comparison with the neglectable efficiency of the commercial WO_3_ powder. It is suggested that the enhanced performance for photocatalytic reduction of CO_2_ with H_2_O to hydrocarbon fuels is attributed to the size-quantization effect-induced change of WO_3_ band gap in this ultrathin 2D nanostructure. In addition, the ultrathin thickness of WO_3_ sheets is also beneficial for the fast charge mobility from the catalyst surface of the reactive agents (for example, CO_2_ and H_2_O) during the photoreduction process.

### Supercapacitors

**Figure 5:**
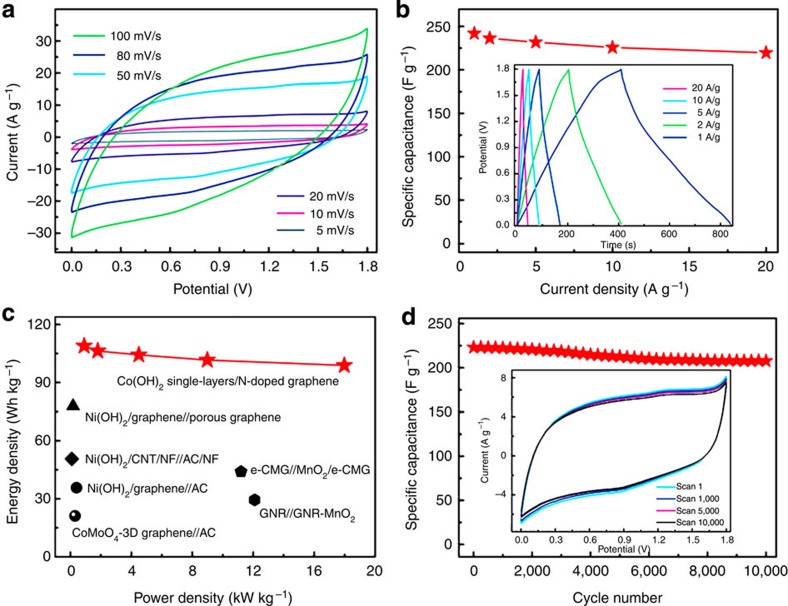
Performance of the *β*-Co(OH)_2_ nanosheet-based supercapacitor. (**a**) Cyclic voltammetry (CV) curves at various scan rates and (**b**) galvanostatic charge–discharge curves at different current densities (inset) and the corresponding calculated specific capacitances of the single-layer *β*-Co(OH)_2_ nanosheet-based all-solid-state asymmetric supercapacitor. (**c**) Comparison of the electrochemical performance with previously reported asymmetric supercapacitors. (**d**) Cycling performance of the fabricated single-layer *β*-Co(OH)_2_ nanosheet-based all-solid-state asymmetric supercapacitor measured at a scan rate of 20 mV s^−1^. Inset: the corresponding CV curves. Reproduced, with permission, from ref. 80 (© 2014 John Wiley & Sons Inc).

Supercapacitor has been recognized as one of the most promising energy-storage devices because of its high power density, fast charging time and long lifetime^86^. It is expected that some ultrathin 2D nanosheets, especially metal hydroxides or oxides, with ultrahigh specific surface area could be attractive electrodes for high-performance supercapacitors^87^. As a typical example, Xie and co-workers reported the fabrication of all-solid-state asymmetric supercapacitor with ultrahigh energy density based on the single-layer *β-*Co(OH)_2_ nanosheet (Fig. 5)^80^. The single-layer *β*-Co(OH)_2_ electrode gave a higher specific capacitance (2,028 Fg^−1^), as compared with the 7-nm-thick *β*-Co(OH)_2_ nanosheet (998 Fg^−1^) and bulk *β*-Co(OH)_2_ electrodes (525 Fg^−1^). The high specific capacitance of single-layer *β*-Co(OH)_2_ nanosheets could be attributed to its ultrahigh specific surface area that enables it with all hydrogen atom-exposed surface. Moreover, the single-layer *β*-Co(OH)_2_ nanosheet was used as the cathode to fabricate all-solid-state asymmetric supercapacitor device, in which the N-doped graphene was the anode. The fabricated cell exhibited specific capacitance of 241.9, 236.3, 231.8, 225.7 and 219.6 Fg^−1^ at the current density of 1, 2, 5, 10 and 20 Ag^−1^ (Fig. 5b), respectively. Importantly, its specific energy density decreased from 108.9 to 98.9 Wh kg^−1^, while the power density increased from 0.9 to 17.98 kW kg^−1^ as the current density increased from 1 to 20 Ag^−1^ (Fig. 5c). Even after 10,000 cycles, its capacitance still remained 93.2% at the scan rate of 20 mV s^−1^ (Fig. 5d), indicating its good cycling stability. Recently, Li and co-workers demonstrated that the ultrathin NiO nanosheets have good performance in supercapacitors^58^. It delivered high specific capacitance of 2,236 Fg^−1^ at 0.5 Ag^−1^. Even at higher current density, the specific capacitance is still maintained, that is, 1,392 Fg^−1^ at 12 Ag^−1^, and 1,576 Fg^−1^ at 4 Ag^−1^ with 99.1% retention after 2,000 cycles, suggesting good rate capability and excellent cycling stability. Although the NiO nanosheets present good performance in supercapacitor, its performance is not as good as the layered *α*-Ni(OH)_2_ nanosheets^58^.

### Photodetectors

**Figure 6:**
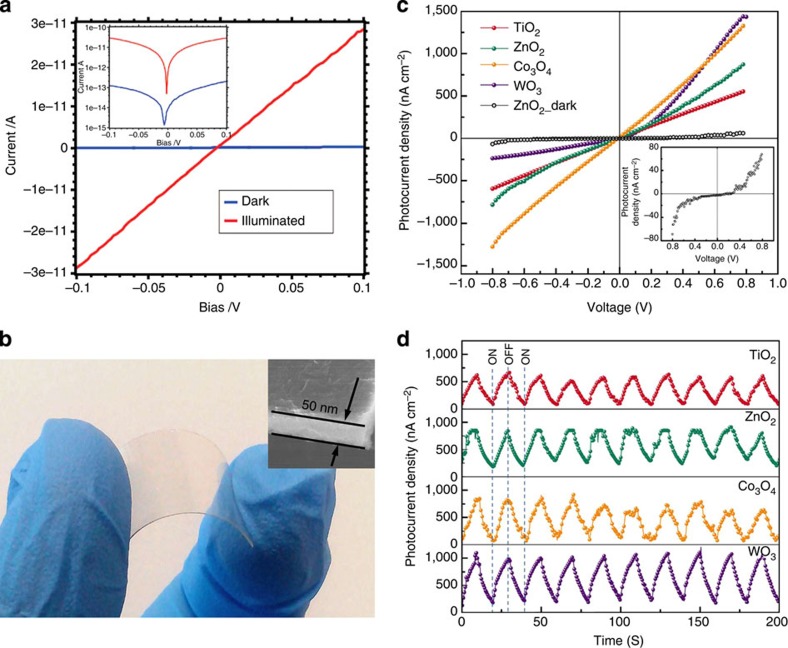
Performance of 2D nanosheet-based photodetectors. (**a**) Current–voltage curves of PbS sheets in dark (red) and under illumination (blue) with a green laser. Inset: current–voltage curves on the logarithmic scale. From ref. 68. Reprinted from permission from AAAS. (**b**) Photograph of the flexibility demonstration of the fabricated electrode. Inset: SEM cross-section image of a typical photoelectrode. (**c**) *I–V* characteristics of photodetectors based on 2D nanosheets of TiO_2_, ZnO, Co_3_O_4_ and WO_3_, respectively. Inset: *I–V* characteristic of the dark photocurrent of 2D ZnO nanosheet photoanode. (**d**) The photoresponse behaviour of photodetectors under illumination with 325-nm ultraviolet light (67 mW cm^−2^) with ON/OFF interval of 10 s and bias of 0.5 V. Adapted from ref. 60.

It was reported that the prepared PbS nanosheets can be readily integrated in a photodetector device without further treatment^68^. The PbS sheet-based device exhibited low conductance in dark without substantial hysteresis (Fig. 6a). The conductance increased by more than two orders of magnitude under illumination of 532-nm laser at intensity of 2.0 mW cm^−2^ (Fig. 6a). A responsivity value of 0.472 AW^−1^ at 0.1 V was achieved. Recently, Dou and co-workers demonstrated that ultrathin 2D metal oxide nanosheets can be used as the active layers to integrate into transparent and flexible ultraviolet photodetectors (Fig. 6b–d)^60^. First of all, the single-layer CVD-grown graphene was transferred on a flexible polyethylene terephthalate substrate as the back electrode. Then, the suspension of ultrathin 2D metal oxide nanosheets in ethanol was spin-coated on the graphene electrode to form transparent and flexible photoelectrode (Fig. 6b). The deposited 2D metal oxide nanosheet layer has thickness of ∼50 nm. The photocurrent responses of the ultraviolet photodetectors were tested under 325-nm ultraviolet light (67 mW cm^−2^) or an alternating ON and OFF ultraviolet source with interval of 10 s. The photocurrent density of the ultrathin 2D metal oxide nanosheet-based photodetectors could reach the order of mA cm^−2^. The WO_3_ photodetector showed a typical *p*-type Schottky barrier contact, while the others exhibited obvious ohmic behaviours with perfect linear current–voltage (*I–V*) responses under the ultraviolet irradiation (Fig. 6c,d). Note that all the ultrathin metal oxide nanosheet-based devices showed much enhanced photocurrent under the ultraviolet irradiation, indicating that TiO_2_, ZnO and Co_3_O_4_ 2D ultrathin nanosheets are promising candidates for fabrication of high-performance and low-cost photodetectors. More importantly, all the devices presented high stability and reproducibility towards the time response. Even after tens of ON/OFF switching cycles, no obvious degradation was detected, proving the excellent stability of the fabricated devices.

### Photothermal therapy

Photothermal therapy is to use the laser irradiation-generated heat to induce hyperthermia within tumour tissues, thus causing the denaturation of proteins, the disruption of cell membrane and subsequent killing of cancer cells^88^. For example, Zheng and co-workers demonstrated that the Pd nanosheet could be a promising photothermal agent for photothermal therapy for cancer^81^. The Pd nanosheets with controllable edge length exhibited the tunable and strong SPR absorption (826–1,068 nm) and high stability on the near-infrared (NIR) irradiation, and those with edge size of 41 nm presented the extinction coefficient of as high as 4.1 × 10^9 ^M^−1 ^cm^−1^ at 1,045 nm. The temperature of 1 ml aqueous solution containing 27-p.p.m. Pd nanosheets could rise from 28.0 to 48.7 °C after 10-min irradiation by a NIR laser (808 nm, 1 W cm^−2^). The significant *in vitro* photothermal cell-killing effect has been achieved by using the Pd nanosheet as the photothermal agent on irradiation of 808 nm laser. More importantly, the Pd nanosheet also exhibited enhanced photothermal stability in comparison with Ag and Au nanostructures with the NIR photothermal effect^81^.

## Future prospects

This review summarizes the recent progress on the wet-chemical preparation and applications of non-layer structured ultrathin 2D nanomaterials. A number of effective wet-chemical methods have so far been developed to prepare non-layer structured 2D nanomaterials (for example, metals, metal oxides and metal chalcogenides) in high yield, large scale and low cost. Excellent performances of these synthesized ultrathin 2D nanosheets for some important applications, especially in catalysis (for example, catalytic organic reactions and CO oxidation), have been demonstrated. From the point of view of the synthesis method, the 2D-templated synthesis method has been proved to be one of the most promising strategies. For example, by using layered or easily prepared 2D nanomaterials as templates, novel non-layer-structured 2D nanomaterials could be prepared through the proper chemical transformation methods such as cation exchange and oxidation. In such processes, the 2D morphology originated from the templating materials could be remained after the chemical transformation. In addition, the hydro/solvothermal synthesis is another appealing approach to synthesize non-layer-structured 2D nanomaterials, in which the experimental parameters, such as the concentration of precursors, solvent, reaction temperature and surfactants, are important factors. Therefore, systematic studies need to be conducted to optimize the experimental conditions to achieve the synthesis of new non-layer structured 2D nanostructures.

Currently, most reported wet-chemical methods are only applicable to synthesize specific materials with uncontrollable size, thickness and crystal phase. Despite that some pioneer work has been conducted, challenges still remain in this field. First, it is difficult to synthesize single-layer nanosheets for most non-layer structured materials by wet-chemical methods. Second, the lateral sizes of most non-layer structured 2D nanomaterials synthesized using wet-chemical methods are less than 1 μm, which is relatively small compared with the nanosheets prepared by exfoliation methods or CVD growth. Last, surfactants are always required during the synthesis process, which are undesirable for some further applications, especially for catalysis and electronic devices. Therefore, it is critical and urgent to find a general and effective way for controlled synthesis of ultrathin non-layer structured 2D nanomaterials. Meanwhile, the mechanisms underlying the crystal growth of ultrathin 2D nanomaterials remain unclear. Therefore, finding simple and efficient techniques for exploration of the mechanism behind these crystal growth processes is also a pressing topic. In addition, most researchers are focusing on producing ultrathin 2D nanomaterials and then demonstrating proof-of-concept applications. Little attention has been paid on the rational design of a material with desired lateral size, thickness, defect, crystal phase and structure. It is well known that the aforementioned structural parameters have significant effect on the performance of a material for specific application. Meanwhile, the favourable structural parameters vary for different applications. Therefore, one of the most challenging fact lies in how we can realize desired performance towards specific application via the rational design of an ultrathin 2D nanostructure with favourable size, thickness, surface state and defect at a highly controllable level.

It is noteworthy that the wet-chemical synthesis of non-layer-structured ultrathin 2D nanomaterials is still in its infant stage. On one hand, there are still a lot of materials whose ultrathin nanosheets have not been synthesized. For example, it is well known that Pt is one of the most robust catalysts for some electrocatalytic reactions (for example, hydrogen evolution and oxygen reduction reaction)^89^,^90^. It is anticipated that ultrathin Pt nanosheets, which have not been synthesized yet, could exhibit excellent catalytic activities for these electrocatalytic processes. Bearing this in mind, one of the future directions is to develop efficient methods for preparation of novel ultrathin 2D nanosheets in single component or multicomponents (for example, alloy), which could possess promising properties and advanced functions. On the other hand, previous studies have demonstrated that the incorporation of other nanomaterials, such as noble metals, metal oxides, semiconductors and polymers, with ultrathin 2D nanosheets (for example, graphene and transition metal dichalcogenide nanosheets) to construct advanced functional composites is a very promising way to further optimize their properties and achieve their superior performances for a wide range of applications^91^,^92^,^93^,^94^,^95^,^96^,^97^,^98^,^99^,^100^. Inspiringly, another promising future direction in this field is the construction of functional hybrid nanomaterials on the basis of these synthesized non-layer structured ultrathin 2D nanosheets to achieve desired performance for numerous applications.

## Additional information

**How to cite this article:** Tan, C. and Zhang, H. Wet-chemical synthesis and applications of non-layer structured two-dimensional nanomaterials. *Nat. Commun.* 6:7873 doi: 10.1038/ncomms8873 (2015).

## Figures and Tables

**Table 1:** Comparison of the typical synthetic methods for 2D nanomaterials.

**Method**	**Brief description of the method**	**Advantage**	**Disadvantage**	**Ref.**
Mechanical exfoliation	Adhesive Scotch tape is used to peel layered bulk crystals. The peeled flakes are then deposit on a target substrate to get single- or few-layer sheets	High quality, large lateral size, few defects, simplicity	Low yield, non-massive production	1,24,25,26
Liquid exfoliation	Direct sonication of layered bulk crystals in solvents or in presence of surfactants or polymers	Solution processibility, massive production, low cost, simplicity	Low yield of single layers, uncontrollable layer number, relatively small lateral size	5,27,28,29,30
Ion-intercalation and exfoliation	Intercalation of layered bulk compounds with Li or Na ions with subsequent sonication in solution	Solution processibility, massive production, high yield of monolayers	Relatively small lateral size with possible defects and phase transformation, the procedure sensitive to water and oxygen	5,31,32,33,34,35
Chemical vapour deposition	One or more volatile precursors react and/or decompose on the exposed substrate surface to produce 2D crystals at high temperature and high vacuum	High quality, large lateral size, controllable thickness, few defects	High temperature, high vacuum, relatively complicated experiments, high cost	36,37,38,39,40
Wet-chemical synthesis	Direct synthesis in the solution phase assisted with surfactants or polymers	Solution processibility, high yield, massive production	Surfactants required, hard to obtain uniform single-layer nanosheets	41,42,43,44,45,46,47,48

2D, two-dimensional.

**Table 2:** List of non-layer structured 2D nanomaterials.

**2D nanomaterial**	**Synthesis method**	**Characterization techniques**	**Dimension (S=size, T=thickness)**	**Application**	**Brief description of the performance**	**Ref.**
Au	2D-templated synthesis	TEM, AFM, XRD	S: 200–500 nm; T: ∼2.4 nm	—	—	51
Au@Ag	2D-templated synthesis	TEM, AFM	S: 100–500 nm; T: 3.0–4.6 nm or 2.8±0.5 nm	—	—	53
Au@Pt, Au@Pd	2D-templated synthesis	TEM, AFM, XRD	S: 100–400 nm; T: 3.5±0.7 nm (Au@Pt)3.4±0.8 nm (Au@Pd)	—	—	54
*α*-Fe_2_O_3_	2D-templated synthesis	TEM, AFM, XRD	S: ∼1 μm; T: 0.55–0.59 nm	—	—	55
CuInS_2_, CuIn_x_Ga_1-x_S_2_, Cu_2_ZnSnS_4_	2D-templated synthesis	SEM, TEM, AFM, XRD	S: 150±40 nm; T: ∼4.8 nm	—	—	56
CuS, CuSe,Cu_2-x_S,Cu_2-x_Se	2D-templated synthesis	SEM, TEM, AFM, XRD	S: 0.6–1.6 μm; T: ∼5 nm	—	—	57
NiO	2D-templated synthesis method	SEM, TEM, XRD, XAFS	S: >1 μm; T: <2 nm	Supercapacitors	The NiO nanosheet-based electrode delivered a high specific capacitance of 2,236 Fg^−1^ at 0.5 Ag^−1^, which still maintained at 1,576 Fg^−1^ at 4 Ag^−1^ with 99.1% retention after 2,000 cycles	58
Rh	Solvothermal method	TEM, AFM, XAFS	S: 500–600 nm; T: <0.4 nm	Organic catalysis	The catalytic activity of Rh nanosheets for the conversion of phenol is four and seven times higher than that of commercial Rh/C and Rh NPs, respectively. Rh nanosheets also exhibited superior catalytic activity and selectivity for the hydroformylation of 1-octene	59
WO_3_, ZnO_2_,TiO_2_,Co_3_O_4_	Solvothermal method	SEM, TEM, AFM, XRD	S: 200 nm (TiO_2_); 1–10 μm (ZnO, Co_3_O_4_ and WO_3_); T: 1.6–5.2 nm	Photodetectors	The photocurrent density of the ultrathin 2D metal oxide nanosheet-based photodetectors could reach the order of mA cm^−2^	60
ZnSe, ZnS	Solvothermal method	TEM, AFM, XRD, XAFS	S: ∼500 nm; T: 0.89–0.92 nm	Photocatalytic water splitting	The single-layer ZnSe-based photoelectrode showed higher photocurrent density compared with all the photoelectrodes based on thick sheets and bulk ZnSe	61
CeO_2_ with surface pits	Hydrothermal method	TEM, AFM, XRD, XAFS	S: >100 nm; T: ∼0.6 nm	CO oxidation	The CeO_2_ nanosheets with surface pits exhibited much lower complete conversion temperature and apparent activation energy for CO oxidation compared with the intact CeO_2_ sheets and bulk CeO_2_	62
Porous In_2_O_3_ with rich oxygen vacancies	Hydrothermal method	TEM, AFM, XRD, XAFS	S: >300 nm; T: 0.88–0.91 nm	Photocatalytic water splitting	Photoelectrode based on In_2_O_3_ porous sheets with rich oxygen vacancies gave a visible light photocurrent of 1.73 mA cm^−2^, which is larger than that of the photoelectrodes based on other In_2_O_3_ materials	63
SnO_2_	Solvothermal method	TEM, AFM, XRD, XAFS	S: >100 nm; T: ∼0.66 nm	CO oxidation	The 0.6-nm-thick SnO_2_ nanosheets exhibited much lower complete conversion temperature and apparent activation energy for CO oxidation compared with the 1.9-nm-thick SnO_2_ sheets, SnO_2_ NPs and bulk SnO_2_	64
Co_9_Se_8_	Solvothermal method	TEM, AFM, XRD	S: >100 nm; T: ∼0.52 nm	—	—	65
Co_9_S_8_-OA	Solvothermal method	TEM, AFM, XRD, XAFS	S: 500–1,000 nm; T: ∼0.5 nm	—	—	66
CdS	Solvothermal method	SEM, TEM, AFM, XRD	S: 300–800 nm; T: ∼4 nm	Photocatalytic water splitting	The CdS nanosheets presented much higher average hydrogen production rate compared with the CdS-DETA hybrid nanosheets and CdS nanosheet-based aggregates	67
PbS	2D-oriented attachment	TEM, AFM, XRD	S: 0.8–2 μm; T: ∼2.2 nm	Photodetectors	The conductance increases by more than two orders of magnitude under illumination of 532-nm laser	68
WO_3_	2D-oriented attachment	TEM, AFM, XRD	S: >1 μm; T: 4–5 nm	Conversion of CO_2_ to CH_4_	The WO_3_ sheets gave good activity towards the reduction of CO_2_ to CH_4_ with H_2_O compared with the neglectable efficiency of commercial WO_3_ powder	70
Au	Assembly of NPs	TEM, AFM,	S: 200–1,000 nm; T: ∼1.68 nm	—	—	71
Eu_2_O_3_	Assembly of nanowires	TEM, AFM, XRD	S: 200 nm-10 μm; T: ∼3.8 nm	—	—	72
PbS	Assembly of nanowires	TEM, AFM	S: 200–250 nm in width and 3–20 μm in length; T: ∼1.8 nm	—	—	73
CdSe	Soft colloidal templated synthesis	TEM, AFM, XRD	S: 200–300 nm in length and ∼100 nm in width; T: ∼1.4 nm	—	—	76
CuS	Soft colloidal-templated synthesis	SEM, TEM, AFM, XRD	S: 453±6 nm; T: 3.2±0.2 nm	Li ion batteries	The CuS electrode exhibited a large discharge and charge capacity of at 0.2 Ag-1, which higher than that of other CuS nanostructures. It also showed good cycling stability	77
SnSe	Colloidal synthesis	SEM, TEM, AFM, XRD	S: 300 nm; T: ∼1 nm	—	—	78
Gd_2_O_3_	Colloidal synthesis	TEM, XRD	S: ∼200 nm; T:<1 nm	—	—	79
*β*-Co(OH)_2_	Liquid-phase synthesis	TEM, AFM, XRD, XAFS	S: 100–400 nm; T: 0.46–0.48 nm	Supercapacitors	The fabricated *β*-Co(OH)_2_-based cell delivered high specific capacitances of 241.9 and 219.6 Fg^−1^ at the current density of 1 and 20 Ag^−1^, respectively. It also showed excellent stability	80
Pd	CO-confined growth	TEM, AFM, XRD	S: 20–160 nm; T: ∼1.8 nm	Oxidation of formic acid	The current density of the Pd nanosheets for formic acid oxidation is ∼2.5 times as active as that of commercial palladium black catalyst	81
				Photothermal therapy	The temperature of aqueous solution with small amount of Pd nanosheets significantly increased under irradiation by a NIR laser	

AFM, atomic force microscopy; 2D, two-dimensional; DETA, diethylenetriamine; NIR, near-infrared; NP, nanoparticle; OA, oleylamine; SEM, scanning electron microscopy; TEM, transmission electron microscopy; XAFS, X-ray absorption fine structure spectroscopy; XRD, X-ray diffraction.
